# Dual roles for a chromatin insulator-H2Av axis in the DNA damage response and hematopoietic development in *Drosophila*

**DOI:** 10.1093/g3journal/jkag185

**Published:** 2026-08-18

**Authors:** James Ryan Simmons, Justin Kemp, Mariano Labrador

**Affiliations:** Department of Biochemistry & Cell and Molecular Biology, The University of Tennessee, Knoxville, TN 37996, United States; Department of Biochemistry & Cell and Molecular Biology, The University of Tennessee, Knoxville, TN 37996, United States; Department of Biochemistry & Cell and Molecular Biology, The University of Tennessee, Knoxville, TN 37996, United States

**Keywords:** insulator proteins, Su(Hw), H2Av, DNA damage response, γH2Av, histone variants, Drosophila hematopoiesis, lymph gland development, genome organization

## Abstract

Chromatin insulator proteins regulate genome architecture and maintain genome integrity, yet how insulator function intersects with the DNA damage response and developmental gene regulation remains poorly understood. Here we show that the *Drosophila* insulator protein Suppressor of Hairy wing [Su(Hw)] and the histone variant H2Av form a functional axis that operates in both contexts. In response to UV and X-ray irradiation, Su(Hw) enrichment at insulator sites increases and Su(Hw) is required for phosphorylation of H2Av, the *Drosophila* equivalent of vertebrate H2AX, establishing Su(Hw) as a component of the DNA damage response. Simultaneous mutation of the damage-sensing kinases ATR and ATM disrupts normal Su(Hw) chromatin binding dynamics, further linking insulator activity to the DNA damage signaling network. Beyond genome integrity, Su(Hw) and H2Av interact genetically to control larval hematopoiesis: mutation of *His2Av* produces melanotic masses and reduced lymph glands, phenotypes that are suppressed in a Su(Hw) dose-dependent manner, while double mutants display supernumerary and overgrown lymph gland lobes. Together, these findings establish dual roles for a chromatin insulator–histone variant axis in which Su(Hw) and H2Av interact to regulate both the cellular response to DNA damage and the transcriptional control of lymph gland development in *Drosophila*.

## Introduction

The activity of a diverse array of DNA-binding proteins is essential for critical chromosomal functions, including transcriptional regulation, cell cycle progression, and the DNA damage response. Histone proteins, which bind DNA directly, play key roles in these processes. This regulatory role is largely due to numerous post-translational modifications that can occur on specific histone residues (Bannister and Kouzarides 2011; Liu et al. 2023). These modifications influence chromatin state, transcriptional output, and the DNA damage response (Jambhekar et al. 2019). Cells also regulate chromosomal activity through histone variants (Szenker et al. 2011; Henikoff and Smith 2015; Jiang and Berger 2017; Giaimo et al. 2019; Martire and Banaszynski 2020; Stevens et al. 2020; Talbert and Henikoff 2021). For example, CENP-A is enriched at centromeric regions, and the mammalian H2A.X is phosphorylated in response to DNA double-strand breaks, signaling to the cell to direct repair machinery to damaged sites (Ward and Chen 2001; Talbert and Henikoff 2010).

A second class of DNA-binding proteins, chromatin insulators, also plays key roles in these processes. Chromatin insulator proteins bind specific DNA sequences throughout the genome and work in pairs or larger groups to regulate the spread of heterochromatin and facilitate interactions between gene enhancers and promoters (Phillips-Cremins and Corces 2013; Schoborg and Labrador 2014; Chen and Lei 2019; Dehingia et al. 2022). In vertebrates, insulator-mediated transcriptional regulation involves loop extrusion, in which the structural maintenance of chromosome proteins cohesin and condensin drive the movement of DNA through the ring complex until encountering an occupied insulator site, thus setting the boundaries of the loops (Fudenberg et al. 2016; Arnould et al. 2021; Gabriele et al. 2022; Morao et al. 2022; Chawla and Csankovszki 2024). Recent work has demonstrated that CTCF functions as a DNA-tension-dependent barrier to cohesin-mediated loop extrusion in mammals (Davidson et al. 2023) However, this loop extrusion mechanism has not been observed in *Drosophila*, where chromatin domains appear to form through alternative mechanisms involving multiple insulator proteins including Su(Hw), BEAF-32, and dCTCF (Matthews and White 2019; Messina et al. 2023; Bhattacharya et al. 2024).

Chromatin insulators also help mitigate the DNA damage response in human cells (Chowdhury et al. 2005; Martin et al. 2014; Ikura et al. 2015; Han et al. 2017; Hilmi et al. 2017; Lang et al. 2017; Sanders et al. 2020; Arnould et al. 2021; Sultana et al. 2025). Previous work by our lab characterized the relationship between the phosphorylated form of the H2A variant in *Drosophila*, γH2Av, and insulator proteins. *Drosophila* H2Av combines the functions of mammalian H2A.X and H2A.Z in one variant that regulates both transcriptional activity and the DNA damage response (van Daal et al. 1988; van Daal and Elgin 1992; Leach et al. 2000; Madigan et al. 2002; Swaminathan et al. 2005; Kotova et al. 2011; Grigorian et al. 2017; Merigliano et al. 2017; Giaimo et al. 2019; Stephenson et al. 2021; Tang et al. 2021; Feng et al. 2022). We observed a close association between Su(Hw) insulator proteins and γH2Av across the genome using immunostaining and ChIP-seq analysis (Simmons et al. 2022). Mutation of any component of the Su(Hw) insulator complex disrupts this interaction, and *su(Hw)* mutants exhibited significantly less γH2Av in polytene chromatin than wild type. Mutation of *su(Hw)* is also associated with spontaneous chromosomal aberrations (CABs) in the actively developing larval brain (Hsu et al. 2020).

The association between insulator proteins and γH2Av was strengthened by treatment with a protein phosphatase 2A (PP2A) inhibitor, suggesting that H2Av phosphorylation promotes a stable association with Su(Hw) insulator complexes (Simmons et al. 2022). Further experiments revealed that γH2Av is a component of insulator bodies that form during osmotic stress and that recovery of these bodies after stress can be prevented by PP2A inhibition. Mutation of *His2Av* suppressed the *ct^6^* phenotype in a sensitized *su(Hw)* heterozygous background, indicating that H2Av and Su(Hw) function in the same pathway (Simmons et al. 2022). An important question that remains unanswered is whether the interaction between Su(Hw) insulator proteins and H2Av is primarily related to the DNA damage response, transcriptional regulation under normal conditions, or both. It is worth noting that these functions may be interconnected, as transcriptional regulation is itself a hallmark of the DNA damage response.

Misregulation of H2Av causes significant developmental phenotypes in larvae. Individuals homozygous for the *His2Av^810^* null allele, or trans-heterozygous for the *His2Av^810^* null allele paired with a chromosomal deficiency lacking the *His2Av* gene (Df(3R)BSC524), develop large melanotic masses at their posterior ends (Grigorian et al. 2017). Homozygous *His2Av^810^* mutants exhibit pupal lethality, highlighting the essential nature of this histone variant. This formation of melanotic masses is correlated with the loss of crystal cells, differentiated plasmatocytes, and undifferentiated prohemocytes from the primary lobes of the lymph gland. It was proposed that H2Av is necessary for prohemocytes to respond to Hedgehog signaling from PSC cells, suggesting an inhibited transcriptional response in prohemocytes (Grigorian et al. 2017). In a separate study, mutation of *His2Av* caused extensive apoptosis in larval eye and wing discs due to misexpression of genes normally regulated by H2Av (Feng et al. 2022). These findings, combined with the known interaction between H2Av and Su(Hw), raise the possibility that insulator function contributes to the gene expression regulation required for normal hematopoietic development.

Based on our previous results showing extensive colocalization of H2Av with Su(Hw) insulator proteins throughout the genome, we investigated whether interactions between H2Av and Su(Hw) are necessary for DNA damage response and whether they influence normal development of the lymph gland. Here, we demonstrate that Su(Hw) and Mod(mdg4)67.2 binding to polytene chromosomes increases in response to UV irradiation. Furthermore, H2Av phosphorylation increases on polytene chromosomes in response to UV and X-ray irradiation, and this increase is reduced in *su(Hw)* mutants. Simultaneous mutation of 2 key DNA damage response kinases, ATR/*mei-41* and ATM/*tefu*, increases Su(Hw) binding to polytene chromatin under native conditions but prevents the increase of Su(Hw) chromatin binding in response to UV irradiation. Our results also reveal a genetic interaction between Su(Hw) and H2Av that influences growth and cell differentiation in the lymph gland. Together, these findings reveal a Su(Hw)-H2Av axis that operates in both the DNA damage response and lymph gland development, raising the possibility that insulator–histone variant interactions coordinate genome maintenance with developmental gene regulation.

## Materials and methods

### Fly stocks and husbandry

All stocks were maintained on a cornmeal agar fly food medium (per liter: 950 mL water, 67.06 g cornmeal, 42.35 g malt extract, 71 mL corn syrup, 15.88 g yeast, 9.18 g soy flour, 5.29 g agar, 4.4 mL propionic acid) supplemented with yeast at 20 °C; crosses were carried out at 25 °C. FlyBase (flybase.org) was used as a reference for gene and allele nomenclature throughout this study (Ozturk-Colak et al. 2024).

The following stocks and alleles are used in this study: The stock *w^1118^*; PBac(RB) *su(Hw)^e04061^*/*TM6B*, *Tb^1^* was obtained from the Bloomington *Drosophila* Stock Center (BDSC: 18224). *su(Hw)^e04061^* is a hypomorphic allele caused by a piggyBac transposon insertion that reduces but does not eliminate Su(Hw) protein. *su(Hw)^V^* is a null allele; stocks carrying this allele were originally obtained from Dr. Victor Corces (Emory University) and are maintained in our lab as *w^1118^; su(Hw)^V^/TM6B, Tb*^1^. Trans-allelic *su(Hw)^V/e04061^* combinations were used to control for second-site mutations. Throughout the manuscript, su(Hw)^−^ denotes loss of su(Hw) function regardless of the specific allele used. *His2Av^810^* is a strong loss-of-function allele originally described as amorphic (Clarkson et al. 1999; Grigorian et al. 2017). The following stocks were provided by our lab: OR, *y^2^w^1^ct^6^*; PBac(RB)*su(Hw)^e04061^*/*TM6B*, *Tb^1^* (derived from BDSC: 18224), *y^2^w^1^ct^6^*; P{Su(Hw)::eGFP}/Cyo; PBac(RB)*su(Hw)^e04061^*/*TM6B*, *Tb^1^*.

### Antibodies

Rabbit polyclonal IgG antibodies against Su(Hw), Mod(mdg4)67.2, and CP190 were previously generated by our lab (Wallace et al. 2010; Schoborg et al. 2013). An antibody against the phosphorylated form of H2Av (UNC93-5.2.1) (Lake et al. 2013) was obtained from the Developmental Studies Hybridoma Bank, created by the NICHD of the NIH and maintained at The University of Iowa, Department of Biology, Iowa City, IA 52242. These antibodies were all diluted 1:1 in glycerol (Thermo Fisher Scientific, BP229-1, lot 020133) and used at a final dilution of 1:200. Secondary antibodies were all diluted 1:1 in glycerol and used at a final dilution of 1:200. The following secondary antibodies were used in this study: Alexa Fluor 594 goat anti-rabbit (Invitrogen, A-111037, lot 2079421), Alexa Fluor 488 donkey anti-rabbit (Invitrogen, A-21206, lot 1834802), Alexa Fluor 488 goat anti-mouse (Invitrogen, A-11001, lot 1858182), and a Texas Red anti-mouse (Jackson Laboratories).

### Immunostaining of larval tissues

Wandering third instar larvae were dissected in phosphate-buffered saline (PBS). Tissues were immediately placed into fixative (4% para-formaldehyde [Alfa Aesar, 43368, lot N13E011], 50% glacial acetic acid [Fisher Scientific, A38-212, lot 172788]) on a coverslip for 1 min. Salivary gland samples were squashed by lowering a slide on top of the sample then turning it over, placing it between sheets of blotting paper, and hitting the coverslip firmly with a small rubber mallet. Slides were dipped in liquid nitrogen, coverslips were removed, and samples were incubated in blocking solution (3% powdered nonfat milk in PBS + 0.1% IGEPAL CA-630 (Sigma-Aldrich, 18896, lot 1043)) for 10 min at room temperature. The slides were dried and incubated with primary antibodies overnight at 4 °C in a box humidified with wet paper towels. The next day, slides were washed twice in PBS + 0.1% IGEPAL CA-630 before incubation with secondary antibodies for 3 h in the dark at room temperature. Slides were washed twice in PBS + 0.1% IGEPAL CA-630, treated with DAPI solution of 0.5 μg/mL (Thermo Fisher Scientific, D1306) for 1 min, and washed one more time in PBS alone.

Samples were mounted with Vectashield antifade mounting medium (Vector Laboratories, H-1000, lot ZF0409), and coverslips were sealed with clear nail polish. All microscopy for immunostaining was performed on a wide-field epifluorescent microscope (DM6000 B; Leica Microsystems) equipped with a 100× (NA 1.35) oil immersion objective and a charge-coupled device camera (ORCA-ER; Hamamatsu Photonics). Image acquisition was performed using SimplePCI (v6.6; Hamamatsu Photonics). Image manipulation was performed in FIJI (Schindelin et al. 2012); all contrast adjustments are linear. Images were further processed in Adobe Photoshop CS5 Extended, Version 12.0 x64. Figures were assembled in Adobe Illustrator CS5, Version 15.0.0. Statistical analyses were performed in GraphPad Prism version 8.0.0 (224) (GraphPad Software, San Diego, CA).

Larval brains were dissected and immunostained using the same protocol as salivary glands. Neurons and neuroblasts were distinguished based on nuclear morphology: neuroblasts were identified by their large, round nuclei, while neurons were identified by their smaller, more compact nuclei. Fluorescence intensity was measured in individual cells using FIJI as described above for polytene chromosomes, with ROIs generated manually for each cell based on the DAPI channel.

### Fluorescence intensity analysis

Images were analyzed for the intensity of each channel (as a proxy for the relative amount of each protein bound to polytene chromatin) using a macro script in FIJI (Schindelin et al. 2012). The DAPI channel was used to automatically generate nonbiased ROIs for each cell, which were then manually curated for extra precision. A rolling-ball background subtraction algorithm (Sternberg 1983) was used for all images. Intensity measurements were made using the measure function. Polytene chromosome images were collected from multiple larvae across independent dissection sessions, with multiple chromosome spreads collected per larva. Each data point in quantification plots represents a single polytene genome. All acquisition parameters were kept constant between slides within each experiment. Because ROIs were generated from the DAPI channel, which specifically labels DNA, the fluorescence intensity measurements reflect signal within the chromatin region rather than the broader nuclear space. Representative images in figures may be cropped for presentation. In some cases, polytene chromosome spreads exceeded the microscope field of view; quantification was performed on the largest contiguous region captured within the DAPI-generated ROI. Pairwise comparisons between 2 groups were performed using Welch's T-tests, which do not assume equal variances. When comparing more than 2 groups, 1-way ANOVA with appropriate post hoc tests was used to control for multiple comparisons. A limitation of this approach is the lack of an internal control protein known to remain unchanged in intensity after genotoxic treatment, which would allow normalization between conditions.

### UV and X-ray irradiation

For UV irradiation, third instar larvae were collected in a small volume of 1× PBS on a glass dissecting dish. Larvae were exposed to 50 mJ/cm^2^ UV irradiation in a UV Stratalinker 2400 (Stratagene) and then allowed to recover for 3 h at room temperature in a vial of fly food before processing each sample. It is worth noting that while UV damage is typically repaired through nucleotide excision repair of pyrimidine dimers rather than double-strand break repair, γH2Av foci have been observed in response to UV irradiation in *Drosophila*, potentially reflecting replication-associated DNA damage or transcription-coupled repair processes (Swaminathan et al. 2005; Joyce et al. 2011). For X-ray irradiation, third instar larvae were collected in a plastic chamber and irradiated at 10 Gy in an RS2000 Biological Irradiator (Rad Source Technologies, Inc.) at a rate of 2 Gy/min. Irradiated larvae were placed in a vial of fly food and larval samples were processed after 3 h of recovery.

### Western blot analysis

Central nervous systems were dissected from 10 wandering third instar larvae per genotype in PBS. Dissected tissues were subjected to X-ray irradiation (10 Gy at 2 Gy/min) using an RS2000 Biological Irradiator (Rad Source Technologies, Inc.). After 30 min of recovery in PBS, tissues were collected in RIPA buffer (150 mM NaCl, 1% NP-40, 0.5% sodium deoxycholate, 0.1% SDS, 50 mM Tris-HCl, pH 8.0) supplemented with protease inhibitor (A32953, Thermo Fisher Scientific) and phosphatase inhibitor (A32957, Thermo Fisher Scientific). Samples were homogenized in a 500 μL PCR tube with a motorized pestle, mixed with 5× loading buffer (0.5 M Tris-HCl pH 6.8, 5% SDS, 0.0125% bromophenol blue, 12.5% β-mercaptoethanol, 50% glycerol) and 15% per volume β-mercaptoethanol, and boiled for 10 min. Protein samples were separated on 12% polyacrylamide gels and transferred onto 0.2 μm PVDF membranes (Immobilon, Millipore). Membranes were blocked in 20% Odyssey Blocking Buffer (LI-COR, 927-50000) in TBS at room temperature for 1 h and then incubated in primary antibodies diluted in TBST at 4 °C overnight. Primary antibodies were mouse anti-γH2Av (UNC93-5.2.1, 1:500; Developmental Studies Hybridoma Bank) and rabbit anti-H2Av (1:5000; Active Motive Catalog No. 39715). Membranes were washed 4 times in TBST for 5 min each, incubated in secondary antibodies diluted in TBST at room temperature for 1 h (goat anti-rabbit 680RD, 1:10,000; donkey anti-mouse 800CW, 1:10,000; LI-COR), washed 4 times in TBST followed by 2 washes in TBS for 5 min each, and imaged on an Odyssey CLx scanner (LI-COR). Images were processed in FIJI (Schindelin et al. 2012).

### Generation of *su(Hw) His2Av* double mutant genotypes

To generate double mutants, flies with mutations of *su(Hw)^e04061^* and *His2Av^810^* in the third chromosome were crossed. Heterozygous F1 females carrying both mutant chromosomes were crossed against the *His2Av^810^*/*TM6B* background, and F2 males were screened for recombinant chromosomes carrying both mutations over the *TM6B* balancer. F2 males were selected for the presence of the *su(Hw)^e04061^* mutation based on eye color and used to establish isogenic lines after 2 more generations of crossing. As a control for potential second-site mutations on the *su(Hw)^e04061^* chromosome, double mutants were also generated using the null *su(Hw)^V^* mutation. Flies with null mutations of *su(Hw)^V^* and *His2Av^810^* were crossed in the background of the *y^2^ct^6^ gypsy* insulator mutations. Heterozygous F1 females carrying both mutant chromosomes were crossed against the *su(Hw)^e04061^*/*TM6B* background, and F2 males were screened for recombinant chromosomes carrying both mutations over the *TM6B* balancer. F2 males were selected for the presence of the *su(Hw)^V^* mutation based on the rescue of the *y^2^ct^6^* mutant phenotype and used to establish isogenic lines after 2 more generations of crossing. All mutant lines were genotyped by PCR to determine the presence or absence of the *His2Av^810^* mutation. The use of 2 independent *su(Hw)* alleles (*su(Hw)^e04061^* and *su(Hw)^V^*) provides evidence that observed phenotypes are due to loss of Su(Hw) function rather than second-site mutations.

### Crystal cell detection and lymph gland phenotyping

Crystal cells in wandering third instar larvae were visualized by incubating between 5 and 10 larvae in 40 μL 1× PBS buffer in a 200 μL PCR tube in a thermocycler set to 70 °C for 10 min. This heat treatment triggers the catalysis of prophenoloxidase enzymes in crystal cells, resulting in cytoplasmic melanin production (Banerjee et al. 2019). Samples were left overnight at 4 °C to increase the intensity of the melanization product for easier detection of crystal cells and lymph glands. Larvae were placed in halocarbon oil 700 (Sigma-Aldrich, H8898) and photographed using a stereomicroscope (MZ16 FA; Leica Microsystems) equipped with a CCD color camera (DFC420; Leica Microsystems). A 150 W white light source (KL 1500 LCD; Leica Microsystems) set to a color temperature of 3,000 K was used for illumination. Images were collected using Leica Application Suite (Version 2.4.0 R1; Leica Microsystems). Image analysis was performed in FIJI (Schindelin et al. 2012) by counting the number of crystal cells visible on the dorsal side of each larva. The frequency at which lymph glands become visible after this treatment was recorded, as were measurements of the number of lobes in each lymph gland and the size of each lobe.

### Sterile wound assay

To determine whether mutant larvae had functional innate immune responses, a sterile wound assay was performed as previously described (Galko and Krasnow 2004). This established assay tests the ability of larvae to form melanized clots at wound sites, which requires functional hemocytes and the melanization cascade. Wandering third instar larvae were selected, washed briefly in 1× PBS buffer and 70% ethanol, and placed on a soft pad constructed of black construction paper (for contrast) taped to a folded-up paper towel. Larvae were stabbed on the dorsal side along the midline approximately between abdominal segments A3 to A4 (Galko and Krasnow 2004) using an insulin syringe needle (Beckton, Dickinson, and Company, Micro-fine IV 28G1/2, 0.36 mm diameter). Larvae were incubated in a vial of fly food overnight at 25 °C before scoring. Larvae that died immediately after stabbing due to unintentional damage were discarded from the count. Note that failure to form a clot can result in larval death due to hemolymph loss.

### Use of artificial intelligence (AI)

AI assistance (Claude Opus 4.6, Anthropic) was used to revise and edit the completed manuscript for concision and clarity.

## Results

### Su(Hw) and γH2Av levels increase in polytene chromosomes after UV irradiation

To determine whether the interaction between chromatin insulator proteins and H2Av is related to the histone variant's role in the DNA damage response, third instar larvae were treated with UV irradiation and allowed 3 h of recovery. Immunostaining of salivary gland polytene chromosomes revealed a significant increase in H2Av phosphorylation in the wild-type OR strain after UV treatment compared to the unexposed control (Fig. 1a, b), consistent with the presence of DNA damage. Additionally, the immunostaining intensity of Su(Hw) protein on polytene chromatin increased significantly after UV irradiation of wild-type larvae (Fig. 1a, b).

**Fig. 1.**
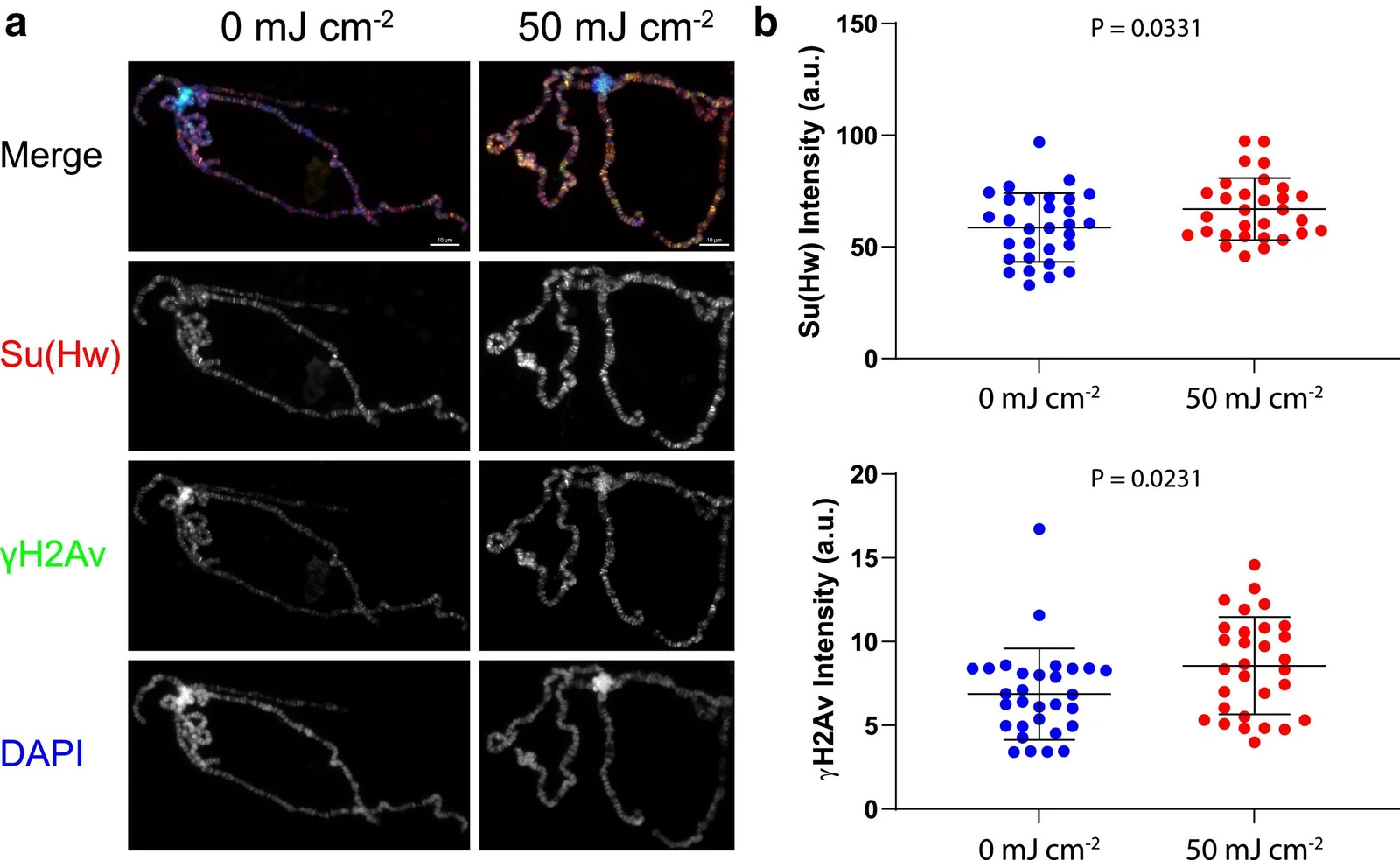
Su(Hw) and H2Av phosphorylation increase in polytene chromatin in response to UV irradiation. a) Representative immunostaining images for Su(Hw) and γH2Av antibodies in polytene chromatin with and without UV treatment. The scale bars are 10 μm each. b) Fluorescent intensities of the Su(Hw) and γH2Av antibody signals in polytene chromatin. Each dot represents an individual polytene genome. *P*-values were determined using unpaired Welch's T-tests. Error bars represent standard error of the mean (SEM).

The level of Su(Hw) protein on polytene chromatin was significantly reduced in the hypomorphic *su(Hw)^e04061^* mutant larvae compared to the wild type by approximately 94% (*P* < 0.0001; Fig. 3a, b). However, Mod(mdg4)67.2, which depends on Su(Hw) for recruitment to chromatin, did not show a statistically significant difference between *su(Hw)^V/e04061^* mutants and wild type (*P* = 0.1988; Fig. 2a, b), despite the known dependence of Mod(mdg4)67.2 on Su(Hw) for chromatin recruitment. Like Su(Hw), Mod(mdg4)67.2 levels increased significantly in polytene chromosomes in response to UV irradiation in wild type (Fig. 2a, b). This increase in Mod(mdg4)67.2 is dependent on Su(Hw), as no significant change in Mod(mdg4)67.2 levels is observed in the *su(Hw)^V/e04061^* mutant after UV treatment (Fig. 2a, b). Intriguingly, increased Mod(mdg4)67.2 staining was observed unbound from the chromatin in the wild type after irradiation. This unbound staining was also noted in the *su(Hw)^V/e04061^* mutant under normal conditions, suggesting that mutation of *su(Hw)* may have similar effects on Mod(mdg4)67.2 localization as DNA damage. However, this phenotype was not observed when *su(Hw)^V/e04061^* mutants were irradiated with UV, implying that the absence of Su(Hw) may drive the accumulation of Mod(mdg4)67.2 in the nucleoplasm and that further DNA damage does not enhance this effect. Although this unbound signal was qualitatively observed in representative images, our DAPI-based ROI approach specifically segments chromatin-associated signal and is not designed to quantify nucleoplasmic protein; therefore, we note this observation descriptively. Notably, the increase in H2Av phosphorylation that occurs in wild-type polytene chromosomes in response to UV irradiation is reduced in the *su(Hw)^V/e04061^* mutant (Fig. 2a, b), suggesting that Su(Hw) is required for the full γH2Av response to DNA damage in salivary gland cells.

**Fig. 2.**
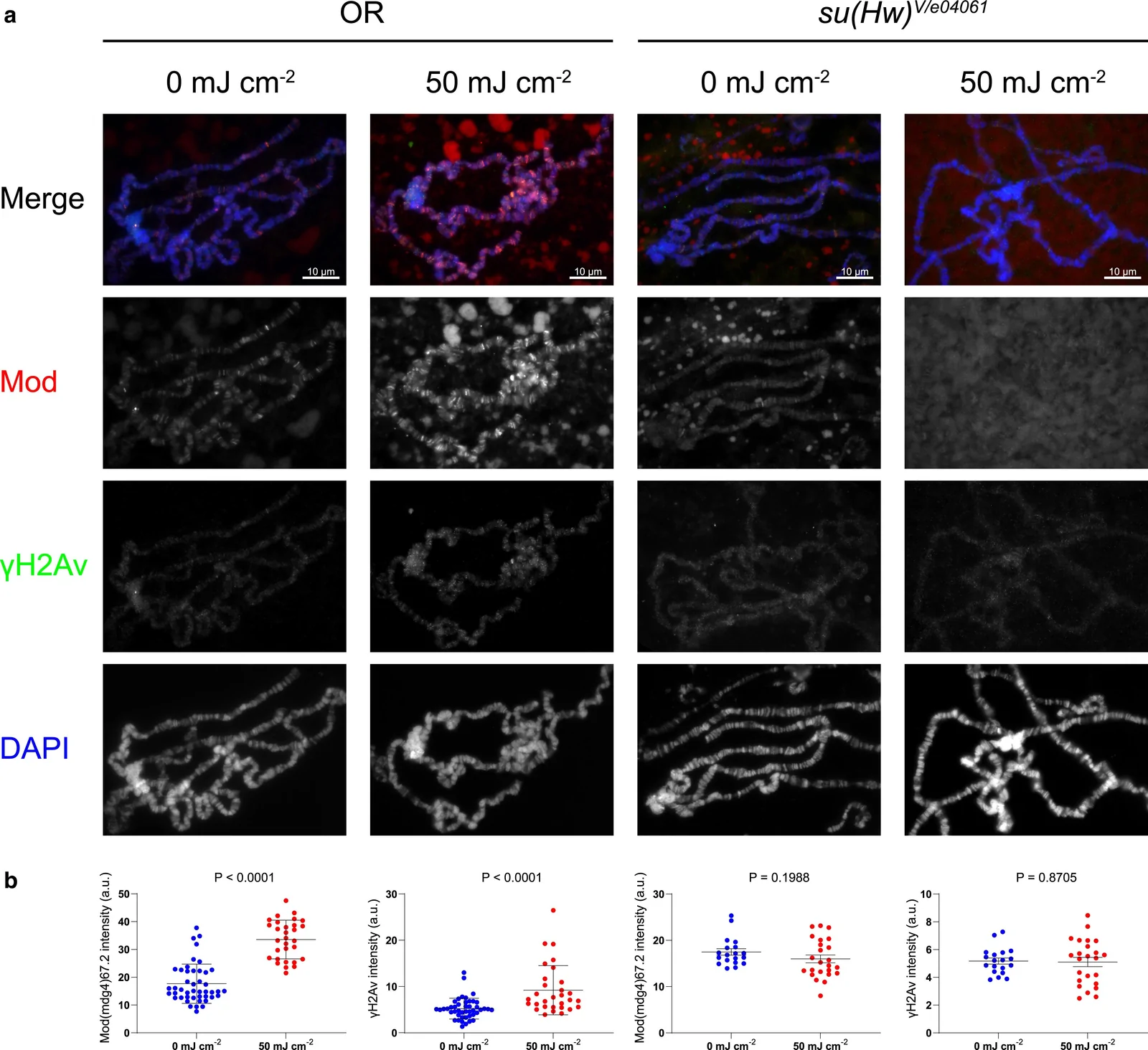
Su(Hw) is associated with H2Av phosphorylation in polytene chromatin after UV irradiation. a) Representative immunostaining images for Mod(mdg4)67.2 (abbreviated as Mod) and γH2Av antibodies on polytene chromosomes with and without UV treatment in wild type (OR) and *su(Hw)^V/e04061^* mutants. The scale bars are 10 μm each. b) Fluorescent intensities of the Mod(mdg4)67.2 and γH2Av antibody signals comparing OR (left graphs) and *su(Hw)^V/e04061^* mutants (right graphs). Each dot represents a single polytene genome. P-values were determined using unpaired Welch's T-tests. Error bars represent standard error of the mean (SEM). Note that y-axis scales differ between OR and *su(Hw)* mutant graphs to accommodate the range of signal intensities in each genotype.

### Su(Hw) is required for the full H2Av phosphorylation response after X-ray irradiation

To determine if Su(Hw) is involved in the cellular response to ionizing radiation, third instar larvae were subjected to a 10 Gy dose of X-rays and allowed to recover for 3 h. Immunostaining in polytene chromosomes showed the expected pattern for Su(Hw), with significantly lower levels in *su(Hw)^e04061^* mutants compared to the wild-type control under both untreated and irradiated conditions (Fig. 3a, b). Unlike the response to UV, the level of nuclear Su(Hw) does not change significantly in polytene chromosomes after X-ray treatment in wild type (Fig. 3a, b). This differential response to UV vs ionizing radiation is notable and may reflect the different types of DNA damage induced by these treatments. UV primarily causes pyrimidine dimers that are repaired through nucleotide excision repair, while ionizing radiation causes a broader spectrum of damage including double-strand breaks, single-strand breaks, and oxidative damage. The increase in Su(Hw) after UV may reflect a transcription-coupled response, whereas the lack of Su(Hw) recruitment after ionizing radiation suggests a different mechanism.

**Fig. 3.**
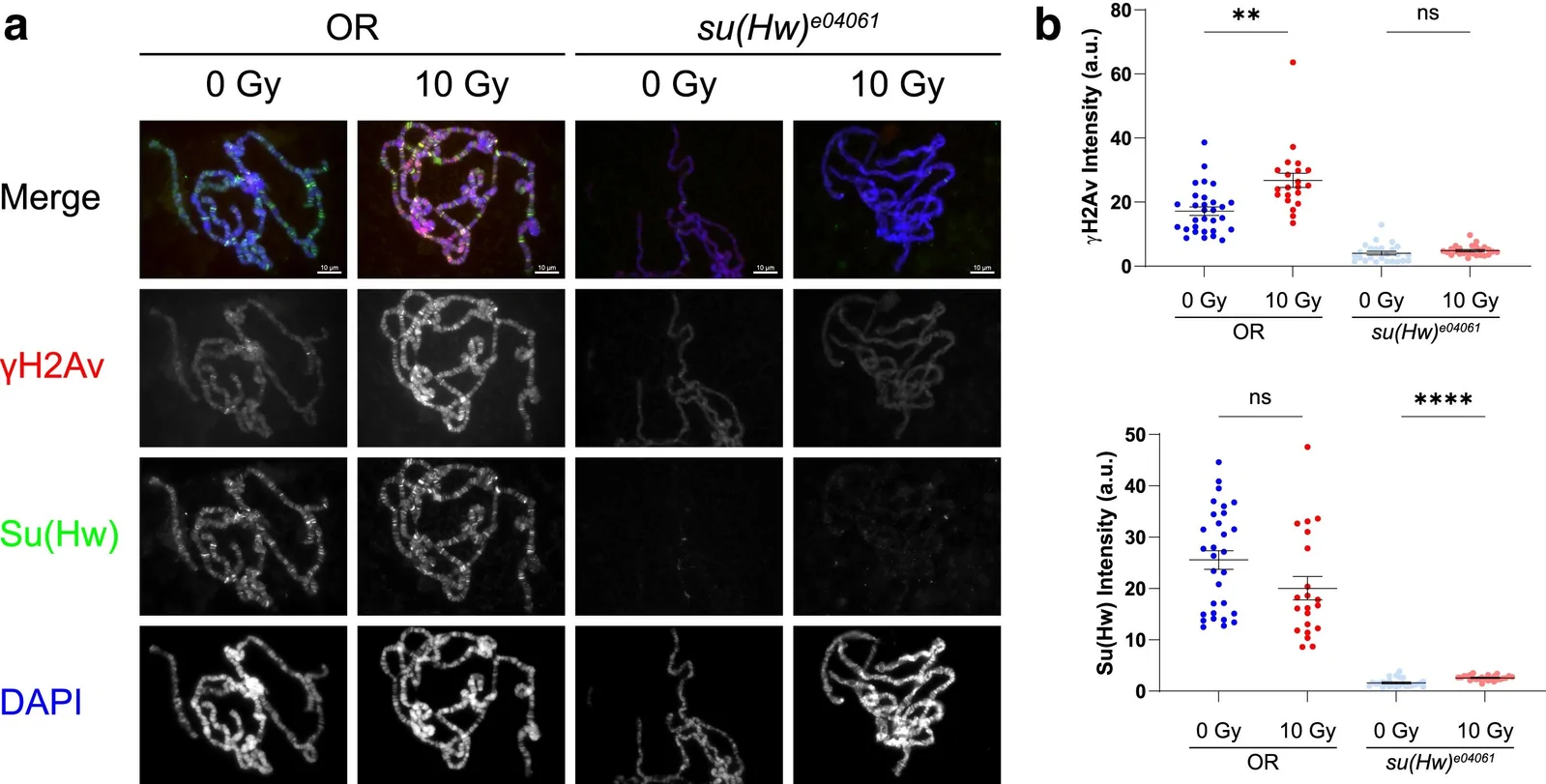
Su(Hw) is associated with H2Av phosphorylation in polytene chromatin after X-ray irradiation. a) Representative immunostaining images for Su(Hw) and γH2Av antibodies in polytene chromatin with and without X-ray treatment. The scale bars are 5 μm each. b) Fluorescent intensities of the Su(Hw) and γH2Av antibody signals. Each dot represents a single polytene genome. P-values were determined using unpaired ANOVA with Dunnett's T3 multiple comparison test where ** *P* < 0.01 and **** *P* < 0.0001. Error bars represent standard error of the mean (SEM).

As expected, the level of H2Av phosphorylation increased significantly in polytene chromosomes after X-ray treatment in wild type (Fig. 3a, b). However, the magnitude of this increase is reduced in *su(Hw)^e04061^* mutants; while γH2Av levels rise after X-ray treatment in both genotypes, the fold increase is smaller in the mutant than in wild type (Fig. 3a, b). This is similar to the response seen after UV irradiation in *su(Hw)^e04061^* mutants, suggesting an association between Su(Hw) and H2Av phosphorylation upon various forms of DNA damage in salivary gland cells. Although different allelic combinations were used for the UV and X-ray experiments, both result in substantially reduced Su(Hw) protein levels, supporting comparison across treatment types. Together these results suggest a mechanism in which Su(Hw) is required for H2Av phosphorylation upon various forms of DNA damage in salivary gland cells, supporting the role of Su(Hw) in the cellular response to ionizing radiation through regulation of H2Av phosphorylation.

To determine whether these findings extend beyond polytene chromosomes to diploid cells, we quantified Su(Hw) and γH2Av immunostaining in larval brain cells after X-ray irradiation, analyzing neurons and neuroblasts separately. Su(Hw) was detected in both cell types, with neuroblasts showing approximately 2.8-fold higher levels than neurons (*P* = 0.0006; Supplementary Fig. 1e). Consistent with the polytene chromosome results, Su(Hw) levels did not increase after 10 Gy X-ray treatment in either neurons or neuroblasts (Supplementary Fig. 1b, c), and Su(Hw) was significantly reduced in *su(Hw)^e04061^* mutants in both cell types (*P* < 0.001; Supplementary Fig. 1b, c). γH2Av levels increased significantly in neurons after irradiation in both wild-type and *su(Hw)^e04061^* mutants (*P* < 0.0001; Supplementary Fig. 1d), and western blot analysis confirmed increased H2Av phosphorylation in whole larval brains after X-ray treatment (Supplementary Fig. 1f). While ChIP-seq analysis would provide higher-resolution mapping of Su(Hw) and γH2Av redistribution after DNA damage, the immunostaining approach used here is consistent with our previous ChIP-seq characterization of Su(Hw)–γH2Av colocalization under native conditions (Simmons et al. 2022).

### Simultaneous mutation of ATR and ATM increases Su(Hw) binding to chromatin

Based on our previous results suggesting that phosphorylation of H2Av stabilizes insulator protein complexes in polytene chromatin, we investigated the effect of mutating the kinases responsible for H2Av phosphorylation. Several kinases have been reported to phosphorylate H2Av, with the *Drosophila* homologs for ATR (*mei-41*) and ATM (*tefu*) being the key examples (Joyce et al. 2011). We therefore examined the effect of mutating these kinases by immunostaining polytene chromatin for Su(Hw). We found no significant difference in the amount of Su(Hw) bound to chromatin in either single mutant (*mei-41^D5^* and *tefu^atm−3^*); however, there was a significant increase (approximately 1.8 fold) in the amount of Su(Hw) in the double mutant (Fig. 4a, b). This unexpected finding—increased Su(Hw) in the absence of both ATR and ATM—may reflect persistent DNA damage that accumulates in the absence of these crucial DNA damage sensors, leading to sustained Su(Hw) localization. Alternatively, the kinases may have roles in regulating Su(Hw) turnover or dynamics at chromatin.

**Fig. 4.**
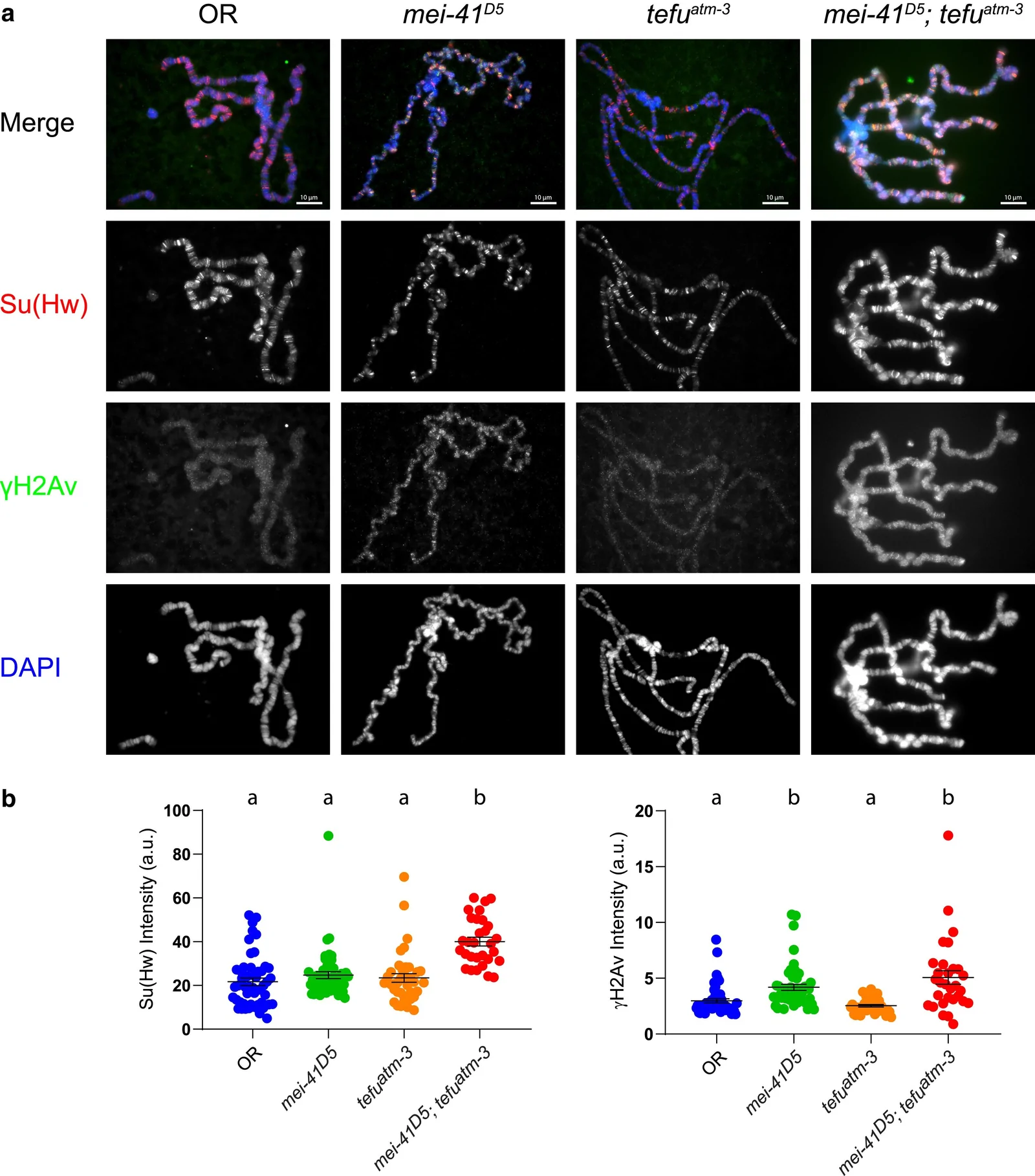
Simultaneous mutation of ATR and ATM increases Su(Hw) bound to chromatin. a) Representative immunostaining images for Su(Hw) and γH2Av antibodies on polytene chromosomes. The scale bars are 10 μm each. b) Fluorescent intensities of the Su(Hw) and γH2Av antibody signals. Each dot represents a single polytene genome. Letters above the graphs represent statistical groupings based on a 1-way ANOVA with Dunn's multiple comparisons test; groups sharing the same letter are not significantly different (*P* < 0.05). Intensities in panel b are absolute fluorescence measurements within DAPI-defined chromatin ROIs; all acquisition parameters were held constant across samples within the experiment. Protein intensities were not normalized to DAPI signal; however, DAPI-normalized values (Su(Hw)/DAPI ratio) were also computed and yield similar or stronger results (2.1-fold enrichment vs 1.8-fold raw), confirming that the observed differences are not driven by variation in chromatin mass. Error bars represent standard error of the mean (SEM). See also Supplementary Fig. 2.

We also measured the effect of these mutations on H2Av phosphorylation in polytene chromatin. We found a significant increase in H2Av phosphorylation in the ATR/*mei-41^D5^* mutant but not in the ATM/*tefu^atm-3^* mutant (Fig. 4a, b). The double mutant also showed significantly more H2Av phosphorylation than wild type, though there was no difference between the double mutant and the ATR/*mei-41* single mutant, suggesting the difference may be primarily attributable to the *mei-41* mutation (Fig. 4a, b). These findings differ from observations in meiotic cells, where more H2Av phosphorylation was observed in the absence of ATM/*tefu* but not ATR/*mei-41* (Joyce et al. 2011) and from mitotic neural cells, which showed decreased frequency of γH2Av foci formation in *tefu^atm-6^* mutants but not *mei-41^29D^* mutants (Merigliano et al. 2017). These discrepancies likely reflect fundamental differences in chromatin organization, cell cycle dynamics and mechanisms of DNA repair between meiotic, mitotic, and polytene cells.

Given the increase in Su(Hw) binding in polytene chromosomes in the double mutant (Fig. 4) and after UV irradiation (Fig. 1), we investigated the response to UV irradiation in these mutants. Su(Hw) immunostaining intensity did not increase after UV treatment in the ATR/*mei-41^D5^* ATM/*tefu^atm-3^* double mutant (Supplementary Fig. 2a, b). The lack of additional increase in the double mutant may be due to the already elevated levels of Su(Hw) observed in this strain under untreated conditions (Fig. 4a, b). H2Av phosphorylation increased in response to UV irradiation in the ATR/*mei-41^D5^* ATM/*tefu^atm-3^* double mutant (Supplementary Fig. 2a, b), suggesting that additional kinases may act redundantly with ATR and ATM in the UV response. Alternatively, the *mei-41^D5^* allele used in this study is hypomorphic rather than null, and residual ATR activity could contribute to the observed γH2Av response after UV treatment.

### Mutation of *His2Av* increases Su(Hw) binding to polytene chromatin

*Drosophila* H2Av is integral to both DNA damage repair and transcriptional regulation. Given our previous exploration of the relationship between Su(Hw) and H2Av phosphorylation in insulator protein mutants, we investigated how insulator activity and binding are affected by mutation of *His2Av*. A heterozygous mutation of *His2Av* in a heterozygous *su(Hw)* mutant background partially rescues defects observed in the wing margins of *ct^6^* mutants (Simmons et al. 2022). Additionally, previous immunostains have shown extensive colocalization between Su(Hw) insulator components and γH2Av (Simmons et al. 2022). Therefore, we examined the binding of insulator proteins to polytene chromatin in the *His2Av^810^* null background.

Quantification of immunostaining signals revealed a modest (approximately 22%) but statistically significant increase in Su(Hw) binding (*P* = 0.01) when comparing *His2Av^810^* homozygotes with *His2Av^810^*/*TM6B* heterozygotes (Fig. 5a). Notably, when normalized to DAPI signal to account for variation in chromatin mass, this increase is more pronounced (approximately 50%), indicating that the effect is not driven by differences in polytene chromosome size between genotypes. While the biological significance of a change of this magnitude is uncertain, it is consistent with the model that persistent unresolved DNA damage in the absence of H2Av leads to sustained Su(Hw) localization. This result may appear contradictory to a simple model in which H2Av and Su(Hw) stabilize each other. However, one interpretation is that in the absence of H2Av, persistent spontaneous DNA damage accumulates and is not properly resolved, leading to sustained Su(Hw) localization. No significant difference was observed in the binding of the other key components of the Su(Hw) insulator complex, Mod(mdg4)67.2 and CP190 (Fig. 5b, c), suggesting that these components are maintained at chromatin through their association with other insulator complexes, such as those containing CTCF. This implies the effect of H2Av mutation on insulator binding to polytene chromatin may be specific to Su(Hw).

**Fig. 5.**
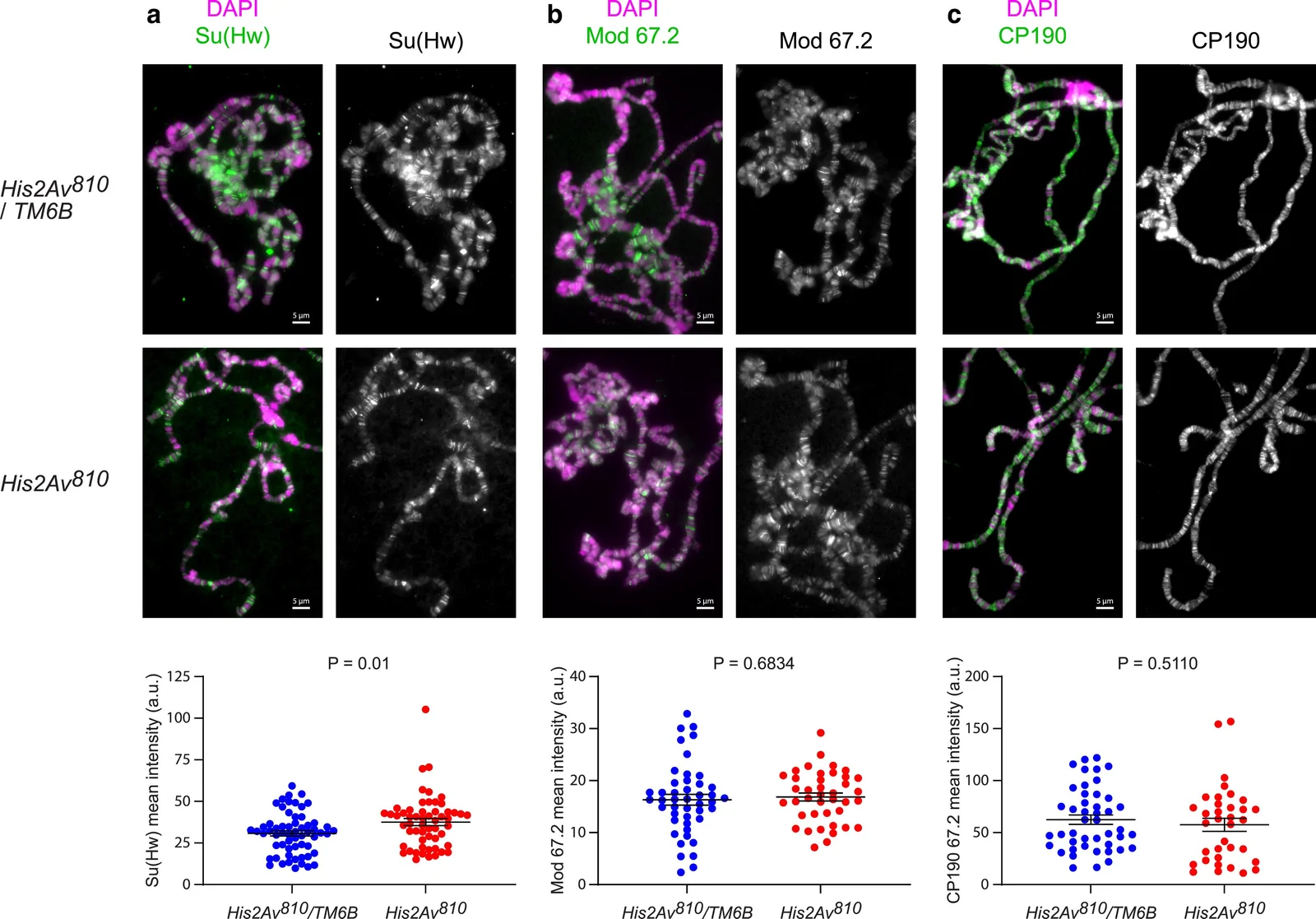
Mutation of *His2Av* increases Su(Hw) binding to polytene chromosomes. Representative immunostaining images and quantification for a) Su(Hw), b) Mod(mdg4)67.2, and c) CP190 antibodies on polytene chromosomes. The scale bars are 10 μm each. Beneath each figure are graphs showing fluorescent intensities of the antibody signals. Each dot represents a single polytene genome. *P*-values were determined using unpaired Welch's T-tests. Error bars represent standard error of the mean (SEM). See also Supplementary Fig. 3.

### Mutation of *su(Hw)* suppresses the *His2Av^810^* melanotic mass phenotype

Our next goal was to determine the effect of mutating insulator proteins on the phenotype of *His2Av* mutants. To explore this relationship, we generated the double mutant genotype *su(Hw)^−^ His2Av^810^* using established mutations of *su(Hw)* (see Materials and methods for details). Double mutant *su(Hw)^−^ His2Av^810^* strains were confirmed by PCR and immunostained for insulator proteins and γH2Av. Staining for Su(Hw), Mod(mdg4)67.2, and γH2Av was significantly reduced in the *su(Hw)^e04061^ His2Av^810^* mutants compared to heterozygous (Supplementary Fig. 3a, b, and e). However, CP190 and CTCF staining were unaffected (Supplementary Fig. 3c, d).

*His2Av^810^* null mutants were recently described to form large melanotic masses in their posterior ends as third instar larvae (Grigorian et al. 2017). Melanotic masses in *Drosophila* can arise from several causes, including aberrant hemocyte differentiation, autoimmune responses, and disrupted lymph gland development. Inspection of the new double mutant *su(Hw)^−^ His2Av^810^* genotypes revealed suppression of the melanotic mass phenotype presented by *His2Av^810^* homozygotes (Fig. 6), implying a genetic interaction between the 2 genes that influences fly hematopoiesis. While most *His2Av^810^* homozygous larvae developed large melanotic masses in their posterior ends, none of the examined *su(Hw)^e04061^ His2Av^810^* homozygous larvae showed such masses. This result was replicated using the independent *su(Hw)^V^* allele in the double mutant *su(Hw)^V/e04061^ His2Av^810^* (Fig. 6), providing evidence that the suppression is due to loss of *su(Hw)* function rather than a second-site mutation on the chromosome. Because *su(Hw)^e04061^* and *su(Hw)^V^* arose independently and carry different molecular lesions, the observation that both suppress the *His2Av^810^* melanotic mass phenotype argues against a linked second-site mutation as the cause of suppression.

**Fig. 6.**
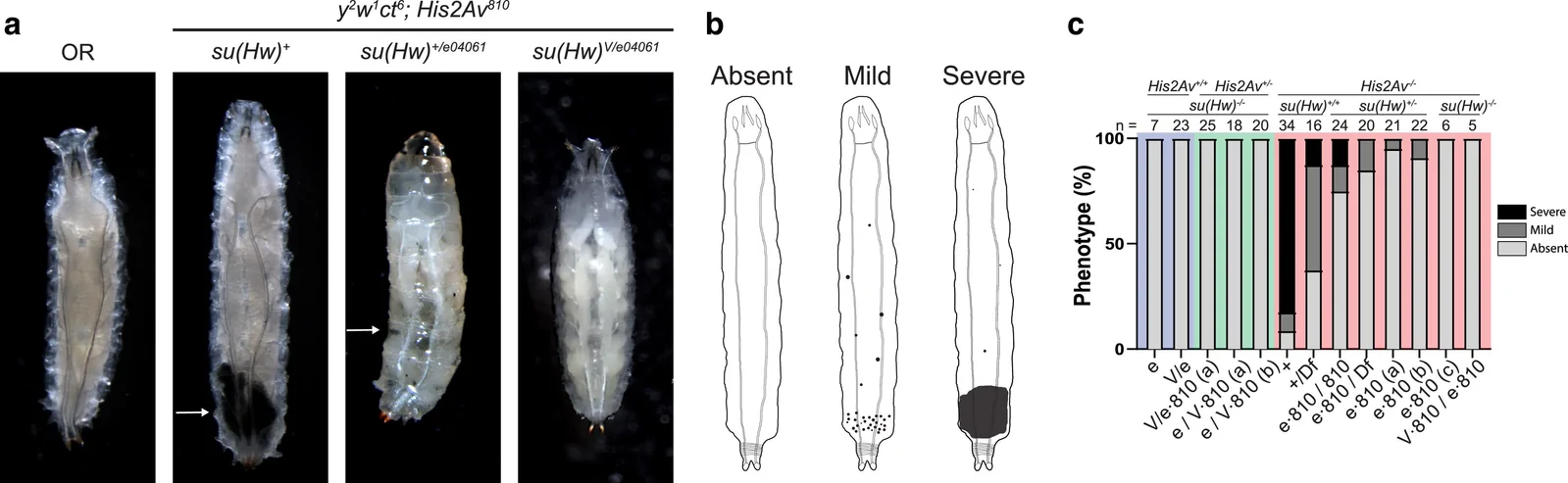
Mutation of *su(Hw)* suppresses the *His2Av^810^* mutant melanotic mass phenotype. a) Representative pictures of select genotypes showing a dorsal view. The presence of black melanotic masses is indicated by white arrows. b) Illustration showing presentation of the melanotic mass phenotype in wandering third-instar larvae (representing each of the phenotypic classes used for melanotic mass scoring: absent, mild, and severe). c) Quantification of melanotic mass phenotype scoring across each genotype examined. Within the bars the light gray color indicates larvae lacking melanotic masses, the dark gray indicates a mild phenotype, and the black represents a severe phenotype. The blue background indicates homozygosity for wild-type His2Av, green indicates heterozygosity for His2Av^810^, and red indicates homozygosity for His2Av^810^. Sample size is shown above each genotype. Genotype abbreviations: e = su(Hw)^e04061^, V = su(Hw)^V^, 810 = His2Av^810^, Df = Df(3R)BSC524. A dot (·) indicates alleles on the same chromosome; a slash (/) separates homologous chromosomes. Letters (a, b, c) denote independent isolates of the same genotype.

Crossing the double mutants against the single *His2Av^810^* mutant allele allowed us to monitor how a partial reduction in the dose of *su(Hw)* affects the formation of melanotic masses in the *His2Av^810^* homozygote. Unlike the double homozygote, *su(Hw)^e04061/+^* heterozygous larvae (in the *His2Av^810^* homozygous background) generated from this cross showed melanotic masses but at a lower frequency than the *His2Av^810^* mutants alone (Fig. 6). These results demonstrate that these masses form in the presence of Su(Hw) and that reducing the amount of Su(Hw) available in the cell is associated with decreased melanotic mass size and frequency in a dose-dependent manner.

### Double mutant *su(Hw)* and *His2Av* larvae exhibit overgrowth of lymph glands

To further characterize the relationship between Su(Hw) and H2Av in the hematopoietic lineage, third instar larvae were subjected to heat treatment at 70 °C. This treatment triggers the catalysis of the prophenoloxidase enzymes in crystal cells, resulting in cytoplasmic melanin production and subsequent darkening of the cells (Banerjee et al. 2019). This provides a convenient method to visualize the numbers and distribution of crystal cells in heat-treated larvae (Fig. 7a). In this illustration, the lymph glands (indicated by arrows) are located along the dorsal vessel near the anterior end of the larva. Heat-treated wild-type larvae show around 50 clusters of crystal cells visible from the dorsal side, while *su(Hw)^e04061^* homozygous larvae display significantly more crystal cell clusters with around 200 visible per larva (Fig. 7b, c).

**Fig. 7.**
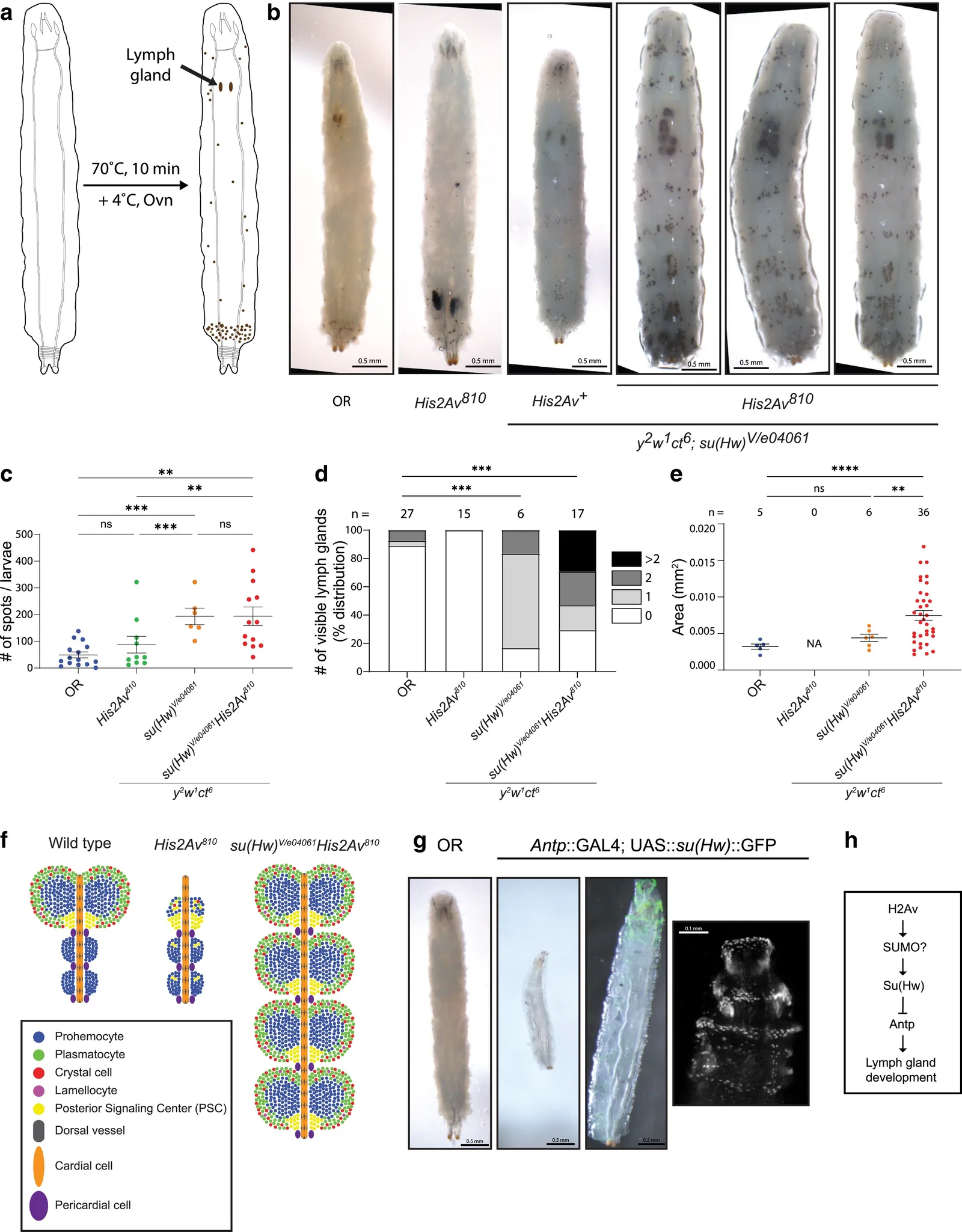
*Su(Hw) His2Av* double mutant larvae exhibit overgrowth of lymph glands. a) Illustration showing the heat treatment assay, which involves incubating larvae at 70 °C and monitoring for melanization of crystal cells and lymph glands. Arrows indicate the position of lymph glands along the dorsal vessel. b) Representative examples of melanized cells and lymph glands. The *y^2^w^1^ct^6^*; *su(Hw)^V/e04061^His2Av^810^* mutant genotype shows larger lymph glands with supernumerary lobes. Scale bars represent 0.5 mm. c) Quantification of melanized spots observed after heat treatment for each genotype. *P*-values were determined using an ordinary 1-way ANOVA with Tukey's multiple comparisons test. d) Number of lymph gland lobes per larva observed in each genotype. *P*-values were determined using pairwise chi-squared tests comparing the distribution of lobe numbers between genotypes. e) Measurements of lymph gland lobe size in different genotypes. *P*-values were determined using ANOVA with Dunnett's multiple comparisons. * *P* < 0.05, ** *P* < 0.01, *** *P* < 0.001, **** *P* < 0.0001. Error bars represent standard error of the mean (SEM). f) Schematic representation of lymph gland organization in wild type, *His2Av^810^*, and *su(Hw)^V/e04061^His2Av^810^* larvae. Note that specific cell type composition within enlarged lymph glands was not determined in this study. g) (i) Wild-type OR larvae for size comparison. (ii) Example of *Antp*::GAL4; UAS-*su(Hw)*::GFP larvae. (iii) Close-up showing brightfield image overlaid with fluorescence from Su(Hw)::GFP observed at the anterior and posterior ends. Spontaneous melanization is observed on the left side of the larvae. (iv) Magnified view of the anterior end of the larvae showing only Su(Hw)::GFP. Scale bars represent 0.5 mm. h) Proposed model for genetic interactions between Su(Hw) and H2Av influencing primary lymph gland development. See also Supplementary Fig. 4.

*His2Av^810^* homozygous larvae show similar numbers of crystal cell clusters as wild type. However, this mutant was reported to have reduced lymph glands and exhibits large melanotic masses containing many crystal cells (Grigorian et al. 2017), complicating the interpretation of this genotype. *su(Hw)^e04061^ His2Av^810^* homozygous double mutant larvae generate significantly more clusters of crystal cells than wild type but a similar number compared to the *su(Hw)^e04061^* single mutant (Fig. 7b, c).

In addition to the circulating crystal cells that become visible upon heat treatment, the lymph glands themselves sometimes become visible due to the presence of crystal cells (Fig. 7a). The frequency of observable lymph glands after heat treatment is significantly higher in the *su(Hw)^e04061^ His2Av^810^* mutants than in wild type (Fig. 7d), likely reflecting both their enlarged size and the greater number of crystal cells producing melanin signal upon heat treatment. Notably, the lymph glands in *su(Hw)^e04061^ His2Av^810^* double mutants were significantly enlarged compared to those in wild-type larvae, often showing supernumerary lobes (Fig. 7b, d). Some larvae in the *su(Hw)^e04061^ His2Av^810^* homozygous mutant displayed up to 8 large lymph gland lobes, and the average size of lymph gland lobes was significantly larger in the *su(Hw)^e04061^ His2Av^810^* double mutant than in wild type (Fig. 7b, d and e). Supernumerary lymph gland lobes were also observed at a low frequency in larvae homozygous for *su(Hw)^e04061^* and heterozygous for *His2Av^810^* (Fig. 7d).

Since immune system development in *Drosophila* larvae largely depends on hematopoiesis in the lymph gland, we tested the innate immune response in these larvae using a sterile wound assay (Supplementary Fig. 4a). This involves stabbing larvae with an ultrafine sterile needle and monitoring surviving larvae for clot formation and melanization at the site of wounding (Galko and Krasnow 2004). This assay revealed that, like the wild-type strain, *su(Hw)^e04061^ His2Av^810^* double mutants are competent to form a clot in response to sterile injury (Supplementary Fig. 4b, c). Statistical analysis confirmed no significant difference in clotting frequency between wild-type and double mutants (chi-squared test, *P* > 0.05). *su(Hw)^e04061^* single mutants also form clots in response to sterile wounds; however, *His2Av^810^* mutants form clots at a slightly lower frequency compared to wild type (Supplementary Fig. 4c), likely due to the previously reported hematopoietic phenotypes (Grigorian et al. 2017). Despite the suppression of the *His2Av^810^* hematopoietic phenotypes observed in the *su(Hw)^e04061^ His2Av^810^* double mutant, mutation of *su(Hw)* in the *His2Av* null background was not sufficient to overcome the pupal lethality associated with the inability to express the essential H2Av histone, with double mutants showing 100% lethality before the end of the pupal stage (data not shown).

Development of the larval lymph gland is influenced by *Antennapedia (Antp)*, which is expressed in the posterior signaling center (PSC), a group of cells at the posterior end of the primary lymph gland that regulates cell fate and differentiation among undifferentiated prohemocytes through paracrine signaling (Krzemien et al. 2007; Mandal et al. 2007). One possibility is that the interaction of Su(Hw) and H2Av regulates the expression of *Antp* in the PSC. To address this possibility, we explored the effect on lymph gland structure and crystal cell production when overexpressing Su(Hw) in cells that normally express *Antennapedia* during larval development. Expression of a UAS-Su(Hw)::GFP construct driven by *Antp*-GAL4 resulted in significant lethality at early developmental stages (data not shown), preventing a direct test of this hypothesis. The few surviving larvae displayed severe growth defects and small melanotic masses (Fig. 7g). Altogether, these data suggest that Su(Hw) and H2Av both influence the developmental pathway that controls the differentiation of hemocytes in the lymph gland of *Drosophila* larvae (Fig. 7h).

## Discussion

Chromatin insulators play an essential role in establishing and maintaining genome architecture during animal development by facilitating long-distance interactions (Maksimenko and Georgiev 2014; Chen and Lei 2019). These interactions help preserve genome integrity against genotoxic stress and regulate gene expression in response to stress or developmental cues. However, the mechanisms linking these activities with insulator function are not fully understood. Our previous research revealed an interaction between the Su(Hw) insulator complex and the H2Av histone variant (Simmons et al. 2022), but whether this interaction contributes to genome integrity, transcriptional regulation, or both remained unclear.

In this study, we demonstrate that the Su(Hw) insulator complex is involved in the DNA damage response in polytene cells. This finding is consistent with our previous results showing that null mutation of *su(Hw)* results in high levels of DNA damage during oogenesis and in the generation of gross CABs in dividing larval neuroblasts (Hsu et al. 2020). This phenotype is common among genes involved in the detection, marking, and resolution of double-strand breaks, such as Aurora A kinase (*aurA*), ATR (*mei-41*), ATM (*tefu*), Twins (*tws*, a PP2A specificity factor), and the topoisomerase gene *Top2* (Gatti and Goldberg 1991; Burma et al. 2001; LaRocque et al. 2007; Despras et al. 2010; Joyce et al. 2011; Mengoli et al. 2014; Mackay and Ullman 2015; Do et al. 2017; Merigliano et al. 2017, 2019). This supports a model where Su(Hw) helps regulate the cellular response to DNA damage.

To test this model, we subjected larvae to UV and X-ray irradiation, which cause different types of DNA damage. Our work shows a significant enrichment of Su(Hw) and Mod(mdg4)67.2, an interacting partner of Su(Hw), on polytene chromatin after UV-induced DNA damage. However, Su(Hw) enrichment does not increase after ionizing radiation. This differential response is notable: UV primarily causes pyrimidine dimers that are repaired through nucleotide excision repair, while ionizing radiation causes a broader spectrum of damage including double-strand breaks, single-strand breaks, and oxidative damage. The increase in Su(Hw) after UV treatment may reflect a transcription-coupled response, as components of nucleotide excision repair in mammalian systems also regulate transcription (Friedberg et al. 2006; Compe and Egly 2016; Sekelsky 2017).

Despite the differential Su(Hw) response to UV vs ionizing radiation, mutation of *su(Hw)* reduced H2Av phosphorylation in response to both UV and X-ray irradiation. This suggests that Su(Hw) is involved in the γH2Av response regardless of the source of damage. However, the mechanism by which Su(Hw) influences H2Av phosphorylation after ionizing radiation, when Su(Hw) itself is not enriched on chromatin, remains unclear, and warrants further investigation.

Our findings on H2Av phosphorylation in polytene cells lacking ATR function (Fig. 4) also differ from findings in meiotic cells in *Drosophila*, which show more H2Av phosphorylation in the absence of ATM/*tefu* but not ATR/*mei-41* (Joyce et al. 2011). Other results in mitotic neural cells show decreased frequency of γH2Av foci formation in the *tefu^atm-6^* mutant but not the *mei-41^29D^* mutants (Merigliano et al. 2017). The increase in Su(Hw) binding to polytene chromatin in the ATR/*mei-41^D5^* ATM/*tefu^atm-3^* double mutant suggests that more Su(Hw) accumulates in chromatin as DNA damage accrues in the absence of these crucial DNA damage sensors, possibly reflecting persistent unrepaired damage. Beyond the kinase question, several lines of evidence support a critical role for H2Av phosphorylation in coordinating the DNA damage response in *Drosophila*. Mutation of *su(Hw)* results in decreased H2Av phosphorylation and formation of spontaneous CABs (Simmons et al. 2022). The CAB phenotype is also observed in *tws* mutants, which are unable to dephosphorylate γH2Av (Merigliano et al. 2017). These results show that regulating the phosphorylation state of H2Av is important for properly initiating and terminating the DNA damage response and that a network of proteins including kinases, phosphatases, and insulator proteins may be involved in this process. Whether Su(Hw) physically interacts with ATR, ATM, or other kinases that phosphorylate H2Av remains to be determined through biochemical approaches such as co-immunoprecipitation.

The finding that Su(Hw) is expressed at significantly higher levels in neuroblasts than in neurons (Supplementary Fig. 1e) suggests that Su(Hw) may have distinct roles in stem vs differentiated cells. This is consistent with the work of Soshnev et al. (2013), who demonstrated that Su(Hw) represses neural genes in non-neural tissues and proposed Su(Hw) as a functional analog of mammalian REST/NRSF, which maintains stem cell identity by repressing differentiation genes (Ballas et al. 2005). The parallel between high Su(Hw) in neuroblasts and high REST in mammalian neural progenitors suggests a conserved logic in which insulator proteins help maintain the undifferentiated state. This may also be relevant to the lymph gland: if Su(Hw) similarly modulates the balance between prohemocyte self-renewal and differentiation, loss of Su(Hw) could alter prohemocyte fate decisions, contributing to the lymph gland overgrowth observed in the double mutants.

Our investigation into the relationship between H2Av and Su(Hw) also revealed significant developmental effects in larval hematopoiesis. We focused on the larval lymph gland, which generates the cells of the circulatory system in *Drosophila*. In larvae, this system comprises differentiated circulating hemocytes in the hemolymph, pockets of sessile hemocytes throughout the animal, and the lymph gland, which generates most hemolymph cells (Grigorian et al. 2017; Banerjee et al. 2019). The lymph gland of a third instar larva consists of 3 sets of lobes that run from anterior to posterior along the dorsal vessel, which acts as the heart by moving hemocytes through the larva (Fig. 7f). The 2 posterior lobes, the secondary and tertiary lobes, are small and generate few hemocytes at this stage. The anterior-most primary lobe is the largest and contains many undifferentiated prohemocytes in addition to differentiated hematocytes, crystal cells, and lamellocytes (Banerjee et al. 2019). Differentiation of prohemocytes is regulated by Hedgehog signaling from cells in the PSC. PSC cell identity is determined by *Antennapedia* expression, the lack of which results in unregulated prohemocyte division in the primary lobe (Banerjee et al. 2019). Melanotic masses, which form in *His2Av* mutants, can arise from aberrant hemocyte differentiation or immune dysregulation and are often associated with lymph gland developmental defects.

Suppression of the melanotic mass phenotype in a Su(Hw) dose-dependent manner is consistent with hematopoietic phenotypes in the *His2Av^810^* mutant resulting from altered *His2Av* expression levels. For example, overexpression of wild-type H2Av results in less cohesion between cells in the PSC and fewer differentiated plasmatocytes and crystal cells (Grigorian et al. 2017). This implies that a precise concentration of H2Av is needed for normal development of this critical tissue. The same is likely true for Su(Hw), which regulates the expression of genes through its role as part of a multi-protein insulator complex whose activity may depend on the relative levels of its components (Mackay and Ullman 2015; Simmons et al. 2022). The finding that single *su(Hw)^e04061^* mutants show more crystal cell clusters than wild type (Fig. 7) implies an H2Av-independent role for Su(Hw) in crystal cell development. An H2Av-dependent function in lymph gland formation is also evident as mutation of *su(Hw)* alone is insufficient to produce the lymph gland overgrowth phenotype. Mutation of *His2Av* alone results in diminished lymph glands, consistent with previous work (Grigorian et al. 2017), whereas mutation of both *su(Hw)* and *His2Av* together generates overgrown lymph glands (Fig. 7d).

An alternative interpretation is that the melanotic mass phenotype of the H2Av mutant depends on the phosphorylated form of H2Av, which in turn depends on Su(Hw) function, as we have previously demonstrated (Simmons et al. 2022). However, evidence suggests that H2Av phosphorylation may not be required for its activity in regulating hematopoietic tissue development (Grigorian et al. 2017). First, expression of a dominant negative mutant allele of *His2Av* lacking the C-terminal 14 amino acid H2AX-like motif (H2AV^CT^) (Clarkson et al. 1999) partially rescues hematopoiesis in the *His2Av^810^* null background and allows larvae to survive to adulthood (Grigorian et al. 2017). Second, overexpression of either the phosphomimetic H2AV^SE^ or the phosphonull H2AV^SA^ alleles partially rescues the phenotype seen in the *His2Av^810^* null background (Grigorian et al. 2017). These results suggest that the role of Su(Hw) in hematopoiesis could be independent of H2Av phosphorylation, pointing instead to a H2Av transcriptional regulatory mechanism (Soshnev et al. 2013; Melnikova et al. 2019). However, interpretation of these rescue experiments is complicated by the finding that increasing the dosage of H2Av proteins, whether wild type or mutant, can itself alter larval hematopoiesis (Grigorian et al. 2017), making it difficult to attribute the rescue specifically to the phosphorylation status of H2Av. H2Av negatively regulates the immune response in flies through modulation of another transcription factor named Relish. This is accomplished through stabilizing SUMOylation levels on Relish, preventing its cleavage and transcriptional activation of downstream genes (Tang et al. 2021). Whether similar regulation is involved in the cooperative activity between H2Av and Su(Hw) remains to be tested (Fig. 7h). Whether Su(Hw) is itself subject to SUMOylation, and how this might influence its transcriptional activity, remains an open question for future experiments.

One possibility to explain the influence of Su(Hw) on H2Av-dependent larval lymph gland development is that Su(Hw) influences *Antp* expression in cells of the PSC, affecting the hedgehog signaling pathway that dictates cellular differentiation in the primary lobes. Our data show that excess Su(Hw) is associated with growth defects, while lack of Su(Hw) in a *His2Av* mutant background causes overgrowth of lymph glands (Fig. 7). It is possible that overexpression of Su(Hw) in *Antp*-expressing cells causes growth defects and spontaneous melanization if Su(Hw) influences *Antp* expression in PSC cells. Alternatively, Su(Hw) may act as a transcriptional repressor (Soshnev et al. 2013; Melnikova et al. 2019), and lack of Su(Hw) in a *His2Av* mutant background may drive aberrant expression of genes involved in stem cell maintenance in prohemocytes, causing overgrowth of lymph glands. Future experiments will investigate the transcriptional output of *Antp* and related genes in these mutant genotypes using approaches such as qRT-PCR. Another hypothesis is that generation of reactive oxygen species in PSC cells, which is involved in lamellocyte maturation (Sinenko et al. 2011), may play a role. If so, this provides another potential mechanism through which Su(Hw) may impact hematopoietic development.

Our results are consistent with a role for Su(Hw) in the DNA damage response, in agreement with previous observations that *su(Hw)* null mutants generate spontaneous CABs (Hsu et al. 2020). We have characterized the relationship of Su(Hw) with the sole H2A variant in *Drosophila*. We previously demonstrated that Su(Hw) and γH2Av extensively colocalize across the genome, though with distinct locus-specific enrichment patterns visible on polytene chromosomes, and that *His2Av* contributes to *gypsy* insulator function in chromatin and insulator bodies (Simmons et al. 2022). We observe reduced H2Av phosphorylation in the absence of Su(Hw); however, lack of H2Av has a modest effect on the recruitment of insulator proteins in chromatin. From these findings we propose a model where H2Av phosphorylation may be stabilized by an intact insulator complex, contributing to the insulator's function. Kinase and phosphatase activity targeting H2Av may regulate insulator complex activity. The most striking manifestation of this interaction is the suppression of the melanotic mass phenotype in *His2Av* mutants. These masses result from developmental defects due to *His2Av* mutation and may not be directly related to the DNA damage response roles of H2Av (Grigorian et al. 2017). The developmental effects of mutating *su(Hw)* and *His2Av* in the larval lymph gland may therefore be unrelated to the DNA damage response, although this possibility has not been ruled out.

Our results suggest that Su(Hw) influences gene expression in the same pathway as H2Av, and mutation of *su(Hw)* and *His2Av* may restore aspects of the expression program in the lymph gland, thereby preventing melanotic mass formation. The dual roles we describe for the Su(Hw)-H2Av axis may reflect a more general principle by which chromatin architectural proteins coordinate mechanisms of DNA damage repair and genome organization with developmental gene expression programs. Insulator proteins like Su(Hw) may therefore serve as nodes that coordinate the structural organization of the genome with both its maintenance and its developmental readout. Whether this integration is a general feature of chromatin insulators, and whether analogous connections exist in vertebrates where CTCF and H2A.X serve related roles, represents an important and largely unexplored question. Together, these findings reveal dual roles for a Su(Hw)-H2Av axis in both the DNA damage response and the transcriptional control of lymph gland development, suggesting that chromatin insulator–histone variant interactions may coordinate genome maintenance with developmental gene regulation.

## Supplementary Material

jkag185_Supplementary_Data

## Data Availability

The authors affirm that all data necessary for confirming the conclusions of the article are present within the article, figures, and tables. Strains and plasmids are available upon request. Supplemental material available at G3 online.
