# Supplementary material for: Dual roles for a chromatin insulator-H2Av axis in the DNA damage response and hematopoietic development in Drosophila
Source: G3 (Bethesda). 2026 Aug 18;16(10):jkag185. doi: 10.1093/g3journal/jkag185 (PMC13643779; doi:10.1093/g3journal/jkag185)
Supplement: Supplementary file 1 [file jkag185_supplementary_data.zip › Figure_S1_G3-2026-406928.pdf]

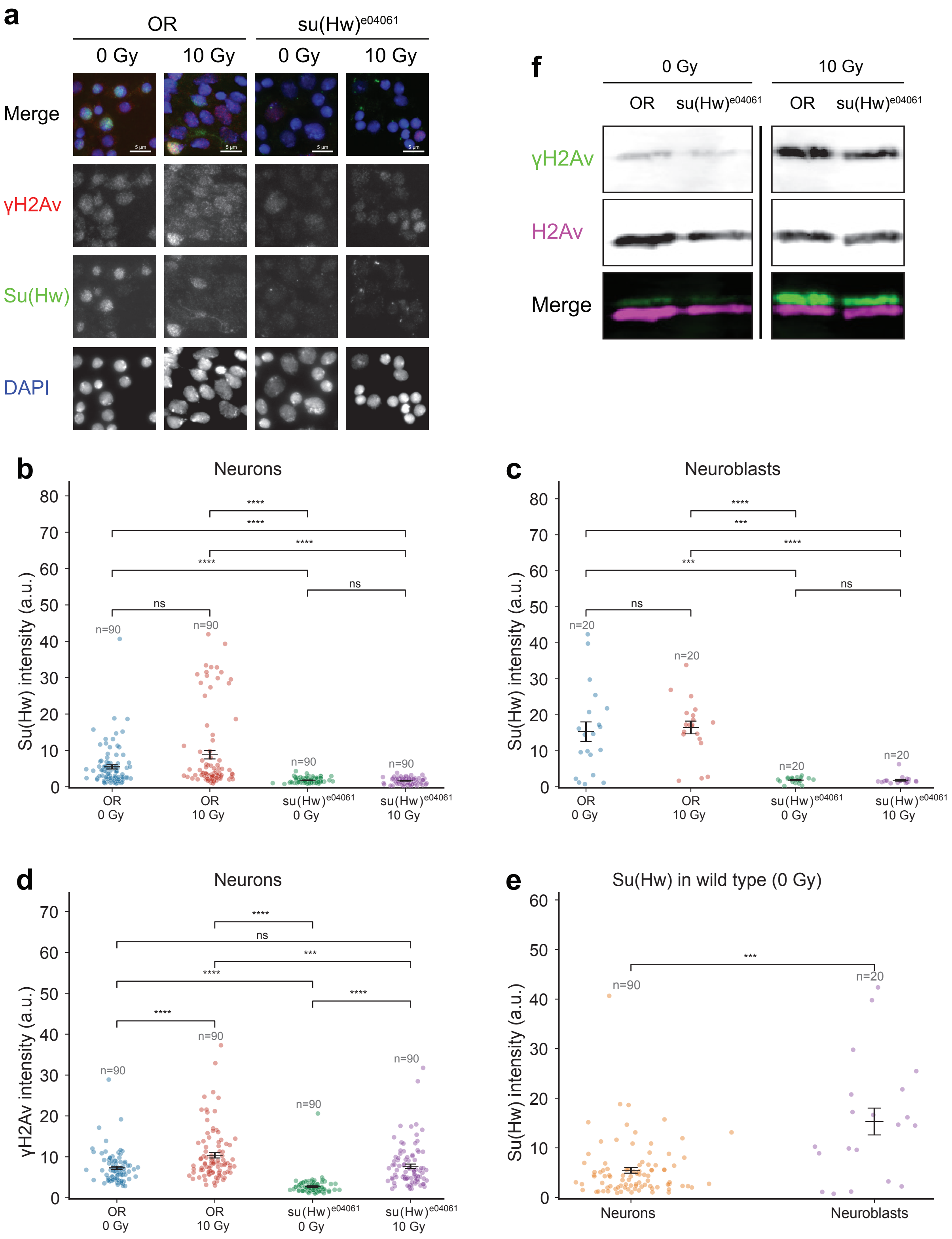

Figure S1. **Su(Hw) and γH2Av in diploid larval brain cells after X-ray irradiation. a**. Representative immunostaining images for Su(Hw) and γH2Av antibodies in larval brain cells from wild type (OR) and su(Hw)e04061 mutant larvae with and without 10 Gy X-ray treatment. Scale bars are 5 μm. **b**. Su(Hw) immunostaining intensity in neurons. **c**. Su(Hw) immunostaining intensity in neuroblasts. **d**. γH2Av immunostaining intensity in neurons. **e**. Comparison of Su(Hw) levels between neurons and neuroblasts in wild type untreated larvae. Neuroblasts show approximately 2.8-fold higher Su(Hw) levels than neurons (P = 0.0006). **f**. Western blot analysis of γH2Av and total H2Av in whole larval brains from wild type and su(Hw)e04061 mutant larvae with and without 10 Gy X-ray treatment. In panels **b-d**, each dot represents a single cell. Error bars represent standard error of the mean (SEM). P-values were determined using Mann-Whitney U tests. Unlike Su(Hw), γH2Av levels did not differ significantly between neurons and neuroblasts (data not shown). Related to Figures 1 and 3.
