# Supplementary material for: Dual roles for a chromatin insulator-H2Av axis in the DNA damage response and hematopoietic development in Drosophila
Source: G3 (Bethesda). 2026 Aug 18;16(10):jkag185. doi: 10.1093/g3journal/jkag185 (PMC13643779; doi:10.1093/g3journal/jkag185)
Supplement: Supplementary file 1 [file jkag185_supplementary_data.zip › Figure_S2_G3-2026-406928.pdf]

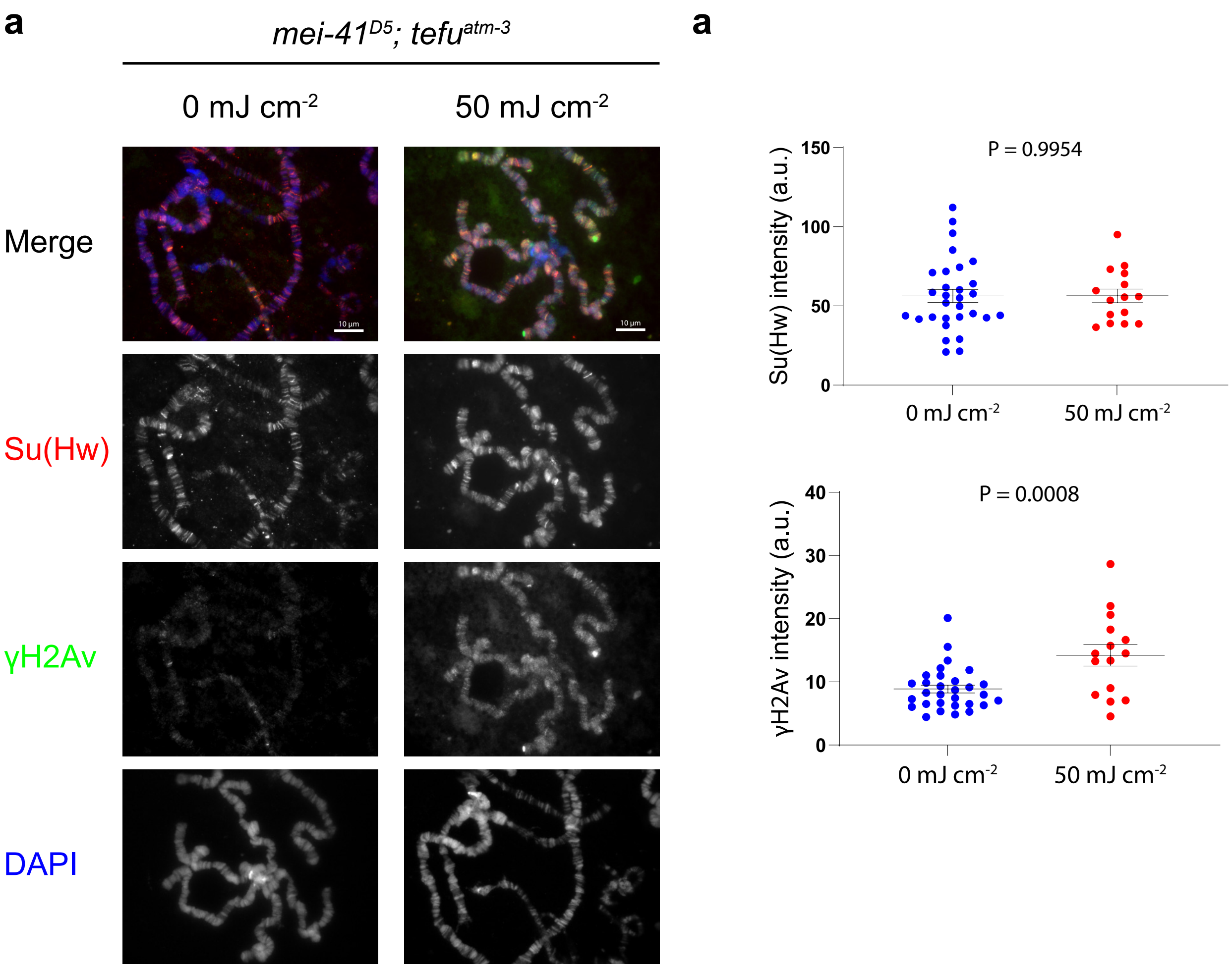

Figure S2. **Su(Hw) enrichment in polytene chromatin after UV irradiation in ATR/ATM mutants**. **a**. Representative immunostaining images for Su(Hw) and γH2Av antibodies on polytene chromosomes with and without UV treatment. The scale bars are 10 µm each. **b**. Fluorescent intensities of the Su(Hw) and γH2Av antibody signals. Each dot represents a single polytene genome. P-values were determined using unpaired Welch's T-tests. Error bars represent standard error of the mean (SEM). Related to Figure
