# Supplementary material for: Dual roles for a chromatin insulator-H2Av axis in the DNA damage response and hematopoietic development in Drosophila
Source: G3 (Bethesda). 2026 Aug 18;16(10):jkag185. doi: 10.1093/g3journal/jkag185 (PMC13643779; doi:10.1093/g3journal/jkag185)
Supplement: Supplementary file 1 [file jkag185_supplementary_data.zip › Figure_S3_G3-2026-406928.pdf]

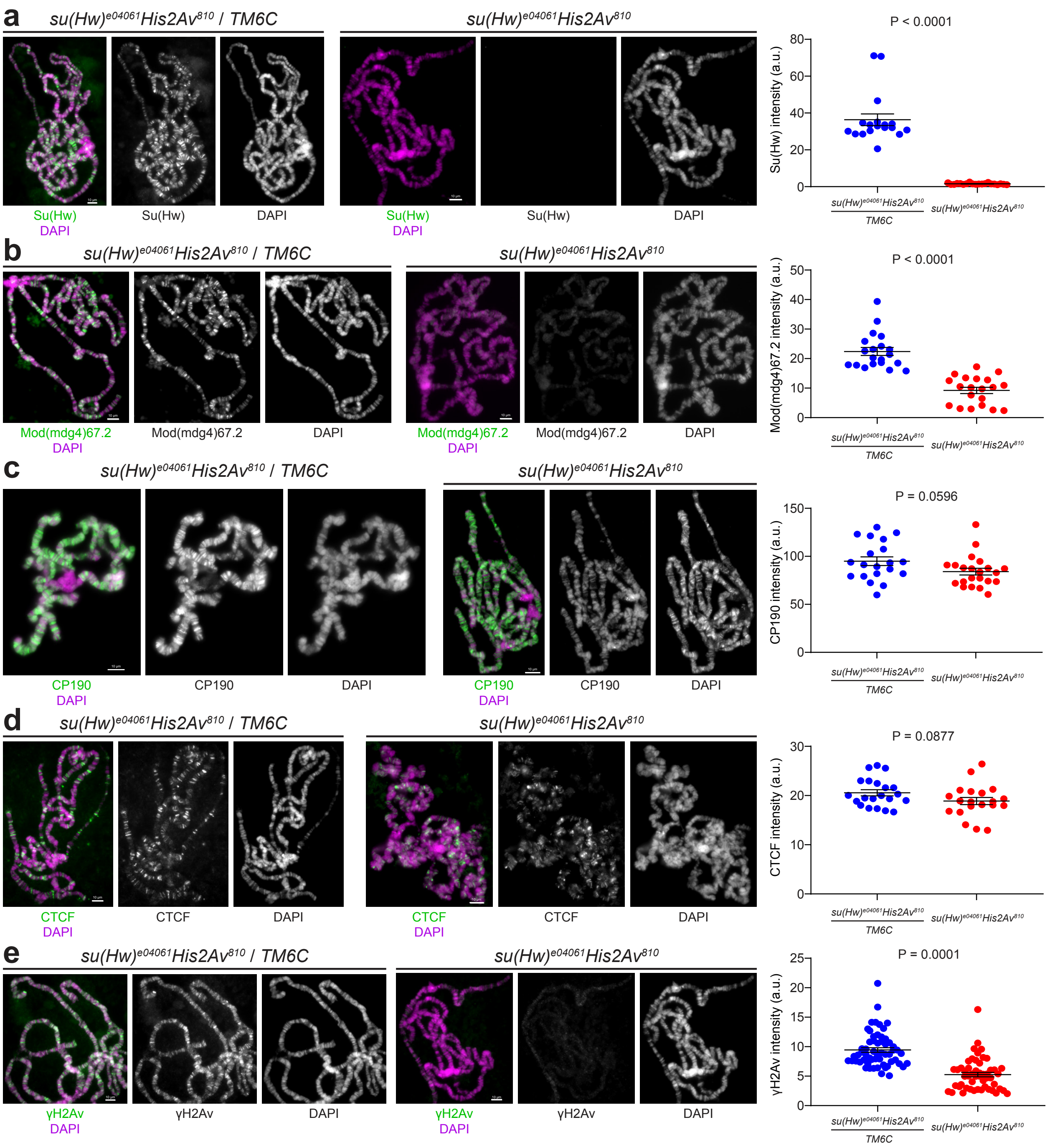

Figure S3. su(Hw) His2Av double mutants show reduced immunostaining of Su(Hw), Mod(mdg4)67.2, and γH2Av. Representative immunostaining images and quantification for **a**. Su(Hw), **b**. Mod(mdg4)67.2, **c**. CP190, **d**. CTCF, and **e**. γH2Av antibodies on polytene chromosomes in su(Hw)e04061 His2Av810 heterozygotes (left) and homozygotes (right). The scale bars are 10 μm each. To the right of each figure are graphs showing fluorescent intensities of the antibody signals. Each dot represents a single polytene genome. P-values were determined using unpaired Welch's T-tests. Error bars represent standard error of the mean (SEM). Related to Figure 5.
