# Supplementary material for: Dual roles for a chromatin insulator-H2Av axis in the DNA damage response and hematopoietic development in Drosophila
Source: G3 (Bethesda). 2026 Aug 18;16(10):jkag185. doi: 10.1093/g3journal/jkag185 (PMC13643779; doi:10.1093/g3journal/jkag185)
Supplement: Supplementary file 1 [file jkag185_supplementary_data.zip › Figure_S4_G3-2026-406928.pdf]

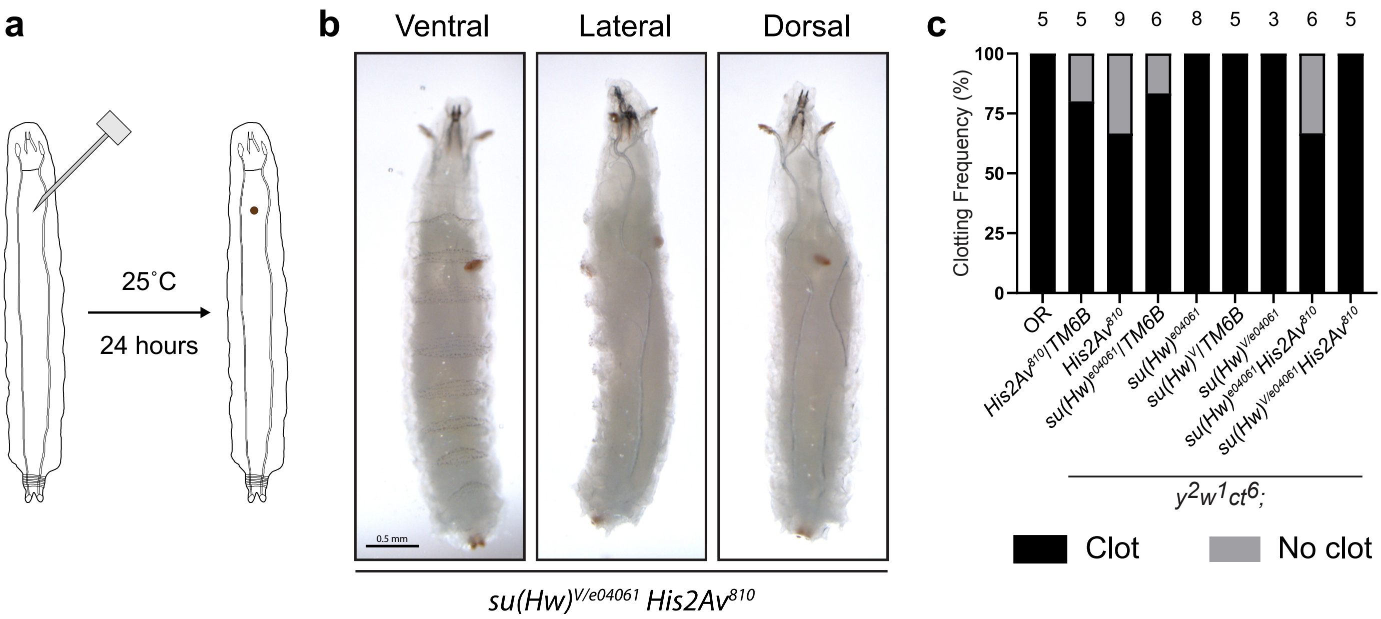


**Figure S4. su(Hw) His2Av double mutant larvae are immunocompetent. a**. Illustration showing the sterile wound assay, which involves stabbing larvae with a fine-tipped needle and monitoring for clot formation. This assay has been previously established to assess hemocyte function in Drosophila (Galko and Krasnow, 2004). **b**. Representative example of clot formation in the y2w1ct6; su(Hw)V/e04061His2Av810 mutant genotype. Views are shown from the ventral, lateral, and dorsal aspects of the same larvae. Two clots are visible, one from the entry wound (dorsal) and one from the exit wound (ventral). **c**. Frequencies of clot formation for different genotypes. Statistical comparison by Chi-squared test showed no significant difference between wild type and double mutant clotting frequencies ($P > 0.05$). Error bars represent stadard error of the mean (SEM). Related to Figure 7.
